# Intraoperative pain management shared decision making with a patient with a complex pain and mental health history: A case report

**DOI:** 10.1016/j.jcadva.2025.100156

**Published:** 2025-08-11

**Authors:** Aubrey Samost-Williams, Jerry Wang, Casey Horani, Eric J. Thomas, Robert J. Volk

**Affiliations:** a Department of Anesthesiology, Critical Care, and Pain Medicine, University of Texas Health Science Center at Houston, Houston, TX, USA; b Department of Internal Medicine, University of Texas Health Science Center at Houston, Houston, TX, USA; c Department of Health Services Research, Division of Cancer Prevention and Population Sciences, MD Anderson Cancer Center, Houston, TX, USA

**Keywords:** Case report, Shared decision making, Pain management

## Abstract

Shared decision making is where the patient and clinician come together to decide on the appropriate course of action in a patient’s medical care based on their goals, preferences, and values. Typically, decision making in anesthesia remains more paternalistic, with clinicians making decisions for patients. However, there is a move towards embracing a shared decision making model. We present here a case report of using shared decision making principles to develop a pain and anxiety management plan in a patient with chronic pain and a complex mental health history. A 40-year-old female with a history of chronic pain, bipolar disorder, generalized anxiety disorder, and postural orthostatic tachycardic syndrome presented for elective temporomandibular joint replacement. Exploration of her prior anesthetic and pain experiences led us to co-create a tailored pain and anxiety management plan drawing on our clinical knowledge and her preferences and experiences. She expressed a high level of satisfaction with pain management and overall anesthetic care. Shared decision making can be a powerful tool in creating perioperative pain management plans. While there are calls for increased use of shared decision making in anesthesia care, most research is in the space of high-risk patients and decisions around anesthetic technique. However, in applying the principles of shared decision making to a lower risk patient’s pain management plan, we were able to better balance pain management, perioperative anxiety, and medication side effects according to her preferences. More research is needed to promote shared decision making throughout anesthesia care.

## 1. Introduction

The care of the patient with a chronic pain history presents unique perioperative pain management challenges. Frequently, there will be multiple *appropriate* options for their anesthetic care during this time, but it can be difficult to decide what is the *best* option for the individual patient. In this situation, it is not uncommon for us as anesthesiologists to make decisions on behalf of the patient grounded in our interpretation of the literature and our prior clinical experiences. [1] This approach to decision making is paternalistic and common, [2] where we as clinicians ultimately do unto the patient what we believe is the best anesthetic.

An alternative approach to paternalistic decision making is shared decision making, where the patient and clinician decide together the best course of action based on the patient’s preferences, goals, and values, which are then combined with the clinician’s knowledge to create a patient-centered care plan. [3–5] For decisions between multiple options that show clinical equipoise, shared decision making is ethically justified based on the principle of autonomy. Shared decision making leverages the privileged information held by the patient, such as their prior experiences with anesthetics, lived experiences in other aspects of healthcare, or fears around the perioperative period. [6] The patient is increasingly recognized as a member of the healthcare team in other healthcare spaces, [7] and despite the patient being unconscious for a large portion of the time we spend caring for them as anesthesiologists, there are opportunities to elevate their voice in the perioperative period. [2,8–10]

In this report, we will share a case of shared decision making with a patient with a complex pain and mental health history.

## 2. Case description

This manuscript follows the CARE case report guidelines. [11,12] Written informed consent was obtained from the patient to present this case.

The patient was a 40-year-old female (59 kg, 173 cm) with a history of bipolar disorder, generalized anxiety disorder, panic disorder, schizoaffective disorder, and postural orthostatic tachycardic syndrome (POTS) presenting for temporomandibular joint (TMJ) replacement for malocclusion. Her home medications included alprazolam and oxcarbazepine for mental health diagnoses, methocarbamol, tizanidine, and pregabalin for dysfunctional TMJ pain, and midodrine for POTS management. Past surgical history was notable for left TMJ replacement 4 months prior to this procedure.

Her prior anesthetic management for the left TMJ replacement appeared uncomplicated upon chart review. Airway management at the time was straightforward with a nasal endotracheal tube placed using video laryngoscopy. She had received 4 mg midazolam as pre-medication. For pain management, she received a dexmedetomidine infusion, ketamine 20 mg bolus with induction, 250 μg fentanyl near the start of the case, and 3 mg hydromorphone intravenously (IV) throughout the 7-h procedure. Per the post-procedure note, she experienced no adverse effects. Therefore, as an anesthesia team, we planned a similar approach to her perioperative management.

We met the patient in the preoperative holding area as operational limitations prevented preoperative clinic evaluation. We opened our discussion by asking generally about her recent contralateral TMJ replacement experience. She displayed a high level of health literacy, with in-depth knowledge of her medications and dosages, which facilitated this conversation. Through this initial open question, we gathered key information that was not immediately apparent in her electronic health record. Specifically, we learned that (1) she has a high tolerance to benzodiazepines, requiring 6–8 mg of alprazolam daily to control her panic disorder and the preoperative midazolam was inadequate for the postoperative anxiety that she experienced in her prior anesthetic; (2) while there was no significant bradycardia documented in the record, the degree of bradycardia relative to her baseline induced by the dexmedetomidine was sufficient to make her symptomatic and was reminiscent of her prior experiences with clonidine in the setting of POTS; and (3) she has a high tolerance to opiates and her impression of pain control from hydromorphone was that it inadequately treated her pain, while ketamine was effective without significant neuropsychiatric side effects.

Given the new information we uncovered in our discussion about her experiences and goals, we were able to propose a new plan. Working in conjunction with her preferences, we repeated 4 mg midazolam IV preoperatively for severe anxiety and added 2 mg of midazolam IV on emergence to cover her postoperative anxiety. Because we would use midazolam to cover the anxiety postoperatively, we decided against dexmedetomidine to avoid the bradycardia and pre-syncopal symptoms that had been distressing in her prior procedure. Given her positive experiences with ketamine, we opted for a higher dose than previously used for pain. We then discussed other opiate options, explaining the risk-benefit profiles of morphine and methadone as potential longer lasting options to replace the hydromorphone that she felt had been less effective. She agreed that the potential benefit of methadone over the first 24 h postoperatively felt most consistent with her goals of sustained pain control, and so we jointly agreed to using methadone. Through this conversation, we learned key information about her clinical experiences and jointly decided on a pain and anxiety management plan that incorporated her preferences and concerns. The entire conversation took approximately 10 min and did not appreciably delay the typical rush of a first-case operating room start. See Table 1 for a summary of the elements of shared decision making and how they were addressed in this case.

Her exam was unremarkable. Her prior TMJ surgical site was well-healed. Her facial movements were symmetric with good mouth opening. Her Mallampati Score was a 2 with a normal thyromental distance and intact neck mobility. Her nares were patent bilaterally. She was visibly anxious but otherwise in no acute distress.

We proceeded to the operating room after 4 mg of midazolam IV, which left her moderately less anxious but fully alert with no evidence of sedation. We induced general anesthesia with 250 μg fentanyl, 150 mg propofol, and 40 mg rocuronium. Mask ventilation was adequate without adjuncts, and a nasal endotracheal tube was placed with video laryngoscopy with a grade 1 laryngeal view on the first attempt. We used sevoflurane for maintenance of anesthesia.

For pain management, we administered 10 mg IV methadone and 20 mg IV ketamine shortly after incision. We gave an additional 30 mg IV ketamine in 10 mg doses every hour and an additional 250 μg fentanyl IV throughout the remainder of the 5-h procedure in response to hemodynamics. She remained stable throughout the procedure. Emergence was smooth and the trachea was extubated when she was awake. Upon extubation, we administered a further 2 mg IV midazolam prophylactically for her history of severe anxiety in the PACU.

She was awake and alert within 15 min of emergence. She rated her pain as moderate, scoring it as a 7 out of 10 on the Numeric Rating Scale, which was satisfactory to her. She was anxious but not experiencing her typical panic disorder symptoms. We expedited access to her partner in the recovery room, which further supported her in controlling her anxiety symptoms. With further pain medications in the Post-Anesthesia Care Unit, her pain level fell to a 4 out of 10 at the time of discharge to the inpatient unit. Table 2 presents a summary of pain and anxiety medications from the day of surgery. She expressed a high level of satisfaction with her perioperative care overall.

## 3. Discussion

Overall, our discussion with the patient dramatically altered our original anesthesia plan. Had we opened our preoperative discussion with the patient presenting our plan as the only or the ideal anesthetic option, we may have unintentionally silenced our patient’s voice and pressured her into an anesthetic that was inconsistent with her desires. [1] “Speaking up” to the authority of a doctor is a barrier to patient’s voicing preferences, [13] and this patient-physician hierarchy is likely compounded by the vulnerability of being in the preoperative area and clothed only in a hospital gown.

Instead of presenting her with our plan conceived from a review of her medical records prior to meeting her, we opened the conversation by asking about her experiences. This allowed her to share significant details about her lived experiences with severe anxiety and panic disorder, as well as experiences of pain perioperatively from a prior TMJ replacement. We were then able to explore with her how she weighed the medication side effects with the potential pain relief. From our perspective as clinicians, these decisions, such as hydromorphone or methadone and midazolam or dexmedetomidine, have relative clinical equipoise in a young otherwise healthy patient. However, from her perspective, there were very clear differences in the efficacy and side effect profiles of these medication options. Therefore, the “best” decision in this case was the one we were able to co-create as a patient-clinician team.

Shared decision making allowed us to use our clinical knowledge to present safe options to the patient from which she could use her experiences and values to decide. Importantly, we presented these options at a patient-appropriate level. We did not make her decide on smaller technical details of her care. Rather, we took her decisions and statements about her values to guide some of these more detailed decisions, such as the exact dosing strategies, on her behalf.

Shared decision making additionally differs from supplying educational decision aids, which might include videos or infographics giving important information to patients around their anesthetic options. [14–16] These help make clinical information accessible to the patient, but if these aids are not coupled with a conversation eliciting the patient’s preferences and values, then they are missing the key contributions of the patient to the shared decision making process. In the case presented here, a preoperative pain management decision aid supplied to the patient alone would not have gotten us to the co-created pain and anxiety management plan that we achieved with our conversation. However, our conversation would likely have been facilitated by a patient education and decision aid, which is unfortunately not currently available at our institution. We were fortunate in this case that our patient had a high degree of health literacy, so we were able to be successful without a decision aid. The shared decision making conversation would have been much harder in someone with a lower degree of health literacy. More research in anesthesia shared decision making aids and implementation of shared decision making processes, especially around perioperative pain management, is needed.

In summary, we presented here a case of shared decision making around perioperative pain and anxiety management. While shared decision making is most important amongst patients at higher perioperative risk, such as patients with multiple co-morbidities or pregnant patients because these anesthetics require tradeoffs between multiple risk, [8] it is increasingly argued that shared decision making should have a role in all anesthetics, regardless of level of risk. [9] This patient was a relatively low anesthetic risk but from her perspective, the overall perioperative experience was demonstrably better because of the shared decision making process. Patients are experts on themselves, and when we empower them to work with us to make decisions, we can capitalize on that expertise. When we fail to actively seek the patient’s voice and learn the information that they hold, we miss out on an opportunity to perform a better anesthetic and increase the quality of care that we provide to our diverse range of patients.

## Figures and Tables

**Table 1:** Essential elements of Shared Decision Making, adapted from Makoul and Clayman, [3] with examples of how they were applied in the care of this patient.

Elements of Shared Decision Making	Application for this Patient
Define and explain the problem	The patient needed surgery to improve her TMJ pain in the long-term, but this was going to cause acute pain initially before improving her chronic pain
Present options	There were many potential options for pain management (morphine, hydromorphone, methadone, ketamine) and anxiety management (midazolam, dexmedetomidine)
Discuss risks and benefits	The risks of each approach were discussed with the patient in detail, with a focus on the most relevant to her, including the impact on pain relief and potential for neuropsychiatric side effects and interaction with her POTS diagnosis
Elicit patient values and preferences	Space was provided in the conversation to allow for her to share her prior anesthetic experiences and preferences based on those experiences
Provide clinical knowledge and/or recommendations	We answered all clinical questions and were sure to present only options that were clinically safe and reasonable given her surgery.
Check for understanding	As the conversation progressed, we checked for her understanding of her options at each step to ensure that she was clear on the tradeoffs that she was making.
Make the decision	We ended the conversation with a clear plan for her pain management and anxiety management, as well as with a clear understanding of her preferences if decisions needed to be made on her behalf intraoperatively.

**Table 2:** Cumulative doses of pain and anti-anxiety medications in the preoperative holding area, the operating room, and the post-anesthesia care unit.

Medication	Total Dose		
	Pre-Operative Holding Area	Operating Room	Post-Anesthesia Care Unit
Anti-Anxiety Medications			
Midazolam IV	4 mg	2 mg	
Alprazolam PO			2 mg
*Opiates*			
Fentanyl IV		500 μg	
Methadone IV		10 mg	
Hydromorphone IV			3 mg
Non-Opiate Anti-Nociceptive Agents			
Ketamine IV		50 mg	
Methocarbamol IV			1000 mg
Dexamethasone IV		6 mg	
Acetaminophen PO			1000 mg
Ketorolac IV			15 mg

## Data Availability

No data was used for the research described in the article.
